# EST2Prot: Mapping EST sequences to proteins

**DOI:** 10.1186/1471-2164-7-41

**Published:** 2006-03-04

**Authors:** Paul Shafer, David M Lin, Golan Yona

**Affiliations:** 1 Department of Computer Science, Cornell University, Ithaca, NY, USA; 2 Department of Biomedical Sciences, Cornell University, Ithaca, NY, USA

## Abstract

**Background:**

EST libraries are used in various biological studies, from microarray experiments to proteomic and genetic screens. These libraries usually contain many uncharacterized ESTs that are typically ignored since they cannot be mapped to known genes. Consequently, new discoveries are possibly overlooked.

**Results:**

We describe a system (EST2Prot) that uses multiple elements to map EST sequences to their corresponding protein products. EST2Prot uses UniGene clusters, substring analysis, information about protein coding regions in existing DNA sequences and protein database searches to detect protein products related to a query EST sequence. Gene Ontology terms, Swiss-Prot keywords, and protein similarity data are used to map the ESTs to functional descriptors.

**Conclusion:**

EST2Prot extends and significantly enriches the popular UniGene mapping by utilizing multiple relations between known biological entities. It produces a mapping between ESTs and proteins in real-time through a simple web-interface. The system is part of the Biozon database and is accessible at .

## Background

Expressed Sequence Tags (ESTs) are partial sequences of cDNA sequences that represent expressed DNA sequences (expressed genes). These short fragments are usually generated by sequencing a few hundred nucleotides from either the 5' end (forward primer) or the 3' end (reverse primer) of a cDNA sequence. Libraries of ESTs can be generated relatively fast and are inexpensive. Therefore, they often serve as a gene discovery tool.

EST libraries are often used to detect genes that are linked with certain diseases or genes specifically expressed in certain tissues. For example, Vasmatzis et al. [1] clustered ESTs using a rudimentary sequence identity metric to discover new genes specific to the human prostate. More recently, Bera et al. [2,3] used a similar procedure to identify MRP8 and MRP9, genes highly expressed in breast cancer. EST libraries were also used to predict, for example, secreted proteins [4].

While EST libraries are potentially very informative, they are difficult to process and analyze. Since ESTs are sequenced by scanning the cDNA only once, they have relatively high error rates due to either sequencing errors (about one sequencing error per 100 residues) or frameshift errors. Thus, nucleic acid sequence databases are flooded with short, redundant and inaccurate or contaminated sequences.

Moreover, ESTs are rarely annotated and the gene protein product is usually undefined. Since ESTs are usually either too short or too noisy for sequence alignment methods to be effective, mapping EST sequences to protein products can be a difficult task without some pre-processing. The signal is often too weak, or the EST might be outside of the translated region. Alternatively, the protein product might not exist in protein sequence databases.

This poses a major problem for experimental molecular biologists who generate large EST libraries to study specific biological systems. A careful investigation of genes is time consuming, therefore only a few of the many EST sequences are usually selected for detailed study. To maximize efficiency, the sequences that are selected are usually ESTs that can be mapped to well-studied genes. With the vast majority of the original EST data ignored, potential advances and new discoveries are limited.

These problems can have a major impact on high-throughput studies. For example, microarrays are commonly used to study a wide variety of biological questions and the ability to correlate differentially expressed genes with a particular experimental manipulation can provide new insight into a given biological problem. However, ESTs with no known function comprise 40–60% of the genes in the genome and therefore many of these differentially expressed genes are likely to be ESTs. Without any further insight into their function, the role of these ESTs in a given biological problem cannot be inferred. Instead, much of the emphasis in analyzing microarray data is devoted to grouping together genes with known function into various categories (e.g. transcription factors, secreted proteins, etc.). As a result, most microarray experiments essentially act to associate known genes with new biological paradigms, and ignore much of the data. Indeed, in recognition of this fact, Affymetrix GeneChip arrays can be ordered that only contain genes of known function, and do not contain ESTs that cannot be annotated.

Similar problems characterize proteomic screens, and the analysis of proteins that are induced or repressed under specific experimental conditions typically focuses only on those with known function. In still another example, genetic screens for mutants will often identify genes that previously had no known function. While these ESTs can now be functionally defined by their observed phenotype, detecting similarity to other proteins with known function can affect the interpretation of the phenotype as well as shape the design of future experiments. In view of these examples, a tool that can associate ESTs with genes of known function would be of great value to biologists looking to understand a given experimental problem.

### Related studies

To increase the effectiveness of EST sequences, one can use clustering procedures. These procedures build upon the high redundancy in EST libraries. The clusters often resolve sequencing errors and are easier to map to protein products than individual ESTs. Three popular databases of EST clusters are UniGene [5,6], TIGR [7] and STACK [8].

Other studies developed models for direct gene prediction from EST data. ESTScan [9] is a program for detecting potential coding regions in EST sequences that is based on a hidden Markov model. Given a nucleic acid sequence, ESTScan uses the Viterbi decoding algorithm to determine the most probable path through the model and to infer the most likely coding region or identify multiple coding regions. The authors report 95% detection rate of true coding regions at about 18% rate of false positives.

DIANA-EST [10] is a program that analyzes ESTs to determine coding regions and frame-shift errors using three different Neural Networks (trained to recognize start codons, coding regions and frameshifts, respectively). The authors report accuracy of about 90% at the nucleotide level. While these methods can be useful for the prediction of coding regions, they do not attempt to map the predicted genes to their protein products. That usually entails additional analysis, BLAST searches and post processing of the search results.

PipeOnline [11] was developed in response to the need for large-scale EST analysis. Users upload raw sequence data which is first cleaned and assembled into a non-redundant set of contigs. PipeOnline then uses BLAST to find protein sequences which are similar to the contigs. ESTAnnotator [12] is another high-throughput EST analysis utility which uses a series of BLAST searches to cluster, assemble, and annotate ESTs. The utility first attempts to annotate the input sequences by querying them against multiple nucleotide databases. If this first step is unsuccessful, ESTAnnotator clusters and assembles the ESTs by iterating applications of BLAST and CAP [13] and the resulting contigs are queried against nucleotide and protein databases. A similar approach is employed in Prot4EST [14] which links together several EST prediction algorithms. It feeds the input sequences through a pipeline of sequence comparisons against various databases starting with rRNA databases (using BLASTN) followed by mitochondrial protein databases (using BLASTX) and other protein databases. If all these comparisons fail to produce significant matches, Prot4EST uses ESTScan or DECODER to annotate the sequence. Another EST analysis package is PartiGene [15] which predicts possible protein products through clustering, sequence comparison and application of other prediction algorithms such as Prot4EST, DECODER, and ESTScan. Also relevant is ESTIMA [16] which is an application designed to assist with EST data management and annotation. The ESTIMA web interface allows users to query their EST data. Users can, for example, find all ESTs associated with a particular GO term, run BLAST queries against the sequences in the database, view contigs and chromatograms, and view BLAST derived annotation.

Current work is largely concerned with identifying gene structure and alternative splicing variants. For example, the latest version of GeneSeqer [17] predicts gene structure (i.e. placement of exons and introns) of a genomic DNA sequence by aligning cDNA and EST sequences to the long DNA sequence and using splice site prediction methods. In addition to these programs there are many other gene prediction programs that are not necessarily geared for EST data and are usually applied to longer nucleic acid sequences or complete genomes (for a review, see [18,19]).

As the discussion above suggests, analysis of EST data is not a simple task. Most EST analysis tools involve a lot of data processing that cannot be done in real time and some require the user to have all the requisite software locally available. This limits the usability of these tools.

In this paper we describe a system that utilizes the Biozon infrastructure [20] and uses EST cluster data together with other data sets, such as established relations between DNA and proteins and similarity data between proteins, to map ESTs to their protein products. Our method is very fast; it uses pre-computed data and does not require intense computations at the query stage. The system can also identify the ESTs whose protein products have specific functions ('target proteins'). In the next sections we describe the main elements of the Biozon's EST2Prot mapping tool and demonstrate its utility in predicting EST sequences whose likely protein products are involved in nerve regeneration.

## Implementation

Biozon's EST2Prot system builds upon the infrastructure of Biozon. It uses multiple data sets, all integrated into a single, tightly connected schema that enables great flexibility in querying for complex relations between entities. Specifically, we utilize the many paths that exist between entities in the Biozon data graph to map ESTs to protein products.

### Biozon

**Figure 1:**
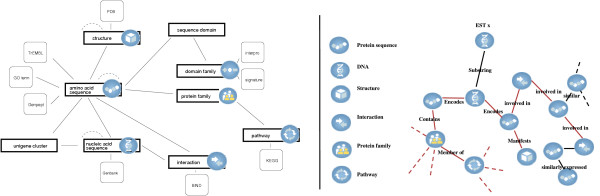
**Left: Partial overview of the Biozon schema**. Biozon currently stores extensive information about more than 50,000,000 objects (integrating sequence, structure, protein-protein interactions, pathways, expression data and more) totaling to about 100 million documents from more than 20 different databases as well as from in-house computations, and 6.5 billion relations between documents (including explicit relations between objects, and derived relations based on different similarity indices). Similarity relations are depicted with dashed lines. The database will be gradually extended to span both new source data types as well well as new computed data. **Right: a subgraph of the Biozon data graph**.

The Biozon database [21] is a system that unifies multiple biological databases consisting of a variety of heterogeneous data types (such as DNA sequences, proteins, interactions, cellular pathways and more) into a single schema. Logically, the database is viewed as a large graph where biological entities correspond to nodes and edges correspond to relations, as is depicted schematically in Figure 1. The underlying assumption of Biozon is that any biological entity or process can be associated with a physical object or a set of physical objects. Therefore, physical objects form the backbone of the database and their physical properties serve as the actual identifiers. For example, a protein is uniquely identified by its amino acid sequence and a DNA by its sequence of nucleotides. An interaction between two proteins or between a protein and a DNA is represented as a *set *of physical objects (the interacting partners), a protein family is a set of protein sequences, a metabolic pathway is a set of reactions (each one associated with a protein (enzyme) family) and so on. Each type of object is also associated with an *identity operator *that is used to compare entities and determine whether they are identical (for example, for sequences the string match operator is used, for sets we use the set-identity operator and for arbitrary subgraphs graph isomorphism is used).

The reliance on physical entities and sets of physical entities as our backbone is especially useful for data integration since it allows unambiguous unification of many entities from different databases based on their physical properties. For example, a protein sequence that exists in Swiss-Prot [22], PIR [23] and RefSeq [24] will be mapped to the same sequence object (node) in the data graph and the information that is available in these sources about this protein will be accessible from a single entry point in Biozon. Unlike identifiers such as accession numbers and cross-references that are potentially unstable or inconsistent (as each database uses its own set of identifiers), relationships that are established based on physical non-redundant Biozon objects are highly reliable and are materialized explicitly in the data graph. This has a great benefit in linking entities from disparate sources. For example, paths are formed between protein domains from InterPro [25] and interactions from BIND [26] or between protein structures from PDB [27] and metabolic pathways from KEGG [28]. Relations between objects in Biozon can have different meanings, depending on the entities they connect. For example, 'member of' is a relation that connects a protein to a protein family or an EST to a EST cluster. The relation 'manifests' relates a protein sequence to its structure, 'encodes' relates a DNA sequence to protein sequence(s), 'similar' relates two similar protein sequences and so on. The large-scale data integration results in a highly connected graph structure that allows one to see each entity in its broader context with all its related entities; a context that cannot be determined from any one source. Utilizing its graph structure, Biozon allows complex and fuzzy searches on the data graph that span multiple data types and specify desired interrelationships between them. For more details on the Biozon schema and its various components see [20].

### Mapping ESTs to proteins

The EST2Prot system exploits a subset of the Biozon schema, including DNA sequences, proteins and EST clusters and the 'encodes', 'substring' and 'similar' relations. We explore five different **direct **paths in the Biozon data graph, and say that EST *s *is *directly mapped *to protein *p *if:

1. *s *encodes *p*

2. *s *is a substring of DNA *s' *near an encoding region of *s' *which encodes for *p *(see section 'Relations').

3. *s *is a member of a UniGene cluster to which NCBI assigns *p*

4. *s *is a member of a UniGene cluster containing *s' *and *s' *encodes *p*

5. *s *is a member of a UniGene cluster containing *s' *and *s' *is a substring of *s" *near an encoding region of *s" *which encodes for *p*

**Figure 2:**
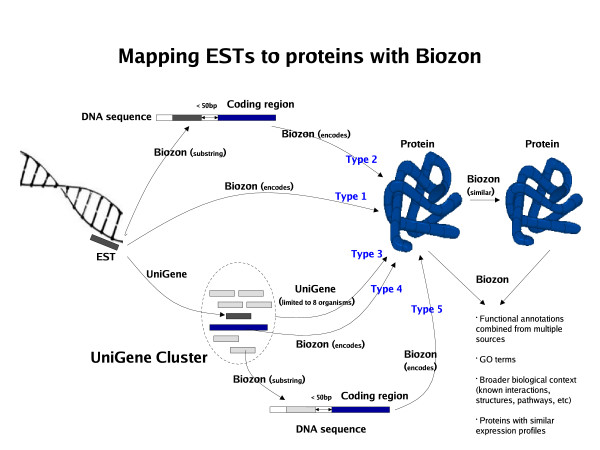
**The Biozon's EST2Prot system**. An EST is mapped to a protein using one of five possible paths. To enrich the set of functional descriptors associated with each EST we also utilize similarity relations between proteins.

We say an EST *s maps *to protein *p *if *s directly *maps to *p ***or **if *s directly *maps to *p' *and *p' *is *similar *to *p *as described in section 'Relations'. An overview of our system is given in Figure 2.

It should be noted that while UniGene relies just on BLAST searches with respect to eight model organisms, Biozon uses all these paths at once to create a more comprehensive mapping between ESTs and proteins. It is the tightly connected schema of Biozon that enables immediate information flow and deduction of paths between entities, without having to resort to external resources outside the database or expensive computations. Most notably, the materialization of similarity data brings forward instantly an unprecedented amount of information that otherwise would requite millions of BLAST searches. This is especially important since often proteins with unknown properties can be characterized based on their similarity with better studied homologous proteins.

#### Data sets

**DNA sequences **are gleaned from GenBank records. As of September 2005 (release 2.2), Biozon contains 42,686,711 unique DNA sequences. **Proteins **are extracted from several databases (including Swiss-Prot/TrEMBL, Genpept, PDB, PIR, BIND and other sources) and unified into a non-redundant set based on their physical sequence of amino acids (rather than based on cross-links). All together, Biozon contains 2,062,061 unique protein sequences in release 2.2.

#### EST clusters

In response to the growing chaos of EST data, NCBI developed UniGene [6], a gene-oriented clustering of transcribed nucleic acid sequences. UniGene includes only protein-coding genes which have at least 100 high quality non-repetitive base pairs. It also requires that its clusters be 3' anchored. Clusters not showing evidence of reaching the 3' terminus are eliminated (these are usually singleton clusters). Each UniGene cluster represents a gene and its alternative splice forms. Associated with each cluster are the gene's possible protein products. These proteins are chosen by comparing the cluster sequences with the available proteomes of eight model organisms [*Homo sapiens *(human), *Mus musculus *(mouse), *Rattus norvegicus *(rat), *Drosophila melanogaster *(fruit fly), *Caenorhabditis elegans *(nematode), *Saccharomyces cerevisiae *(baker's yeast), *Escherichia coli*, and *Arabidopsis thaliana *(mouse-ear cress)]. For each model organism, the cluster is assigned the protein most similar to a representative sequence with respect to some similarity threshold (BLAST evalue less than 1e-6). If no sequence in a cluster has a significant BLAST match, then that cluster is left unassigned. In fact, UniGene do not assign proteins to 42% of its clusters. UniGene clusters for 54 organisms were integrated into the Biozon schema and in release 2.2 this dataset contains 807,175 clusters with a total of 19,471,927 EST sequences.

#### Relations

To determine possible links between EST sequences and proteins we explore several paths, as is depicted in Figure 2. These paths are based on the following relations.

##### The 'encodes' relation

This relation ties nucleic acid sequences and proteins. The relations are not established based on cross links, but rather based on physical properties. Each encodes relation (*d*, *p*) indicates that the DNA sequence *d *contains a coding region that can be translated completely to the protein sequence *p*.

##### The 'UniGene encodes' relation

This relation is established between UniGene clusters and proteins. The relations are established by the UniGene team as described above.

##### The 'substring' relation

**Figure 3:**
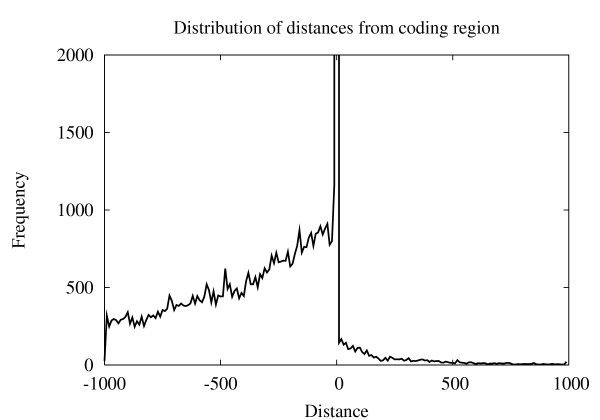
**Mapping ESTs to proteins through the substring relation**. Often, a nucleic acid sequence is a fragment of a longer DNA sequence that contains a coding region. We compared all mouse nucleic acid sequences to each other and studied the distribution of (minimal) distances from coding regions. The vast majority of fragments (250,000) are located at the beginning of a coding region of a longer DNA sequence. In addition, there is a substantial number of ESTs that are located in the proximity of a coding region.

This relation exists between strings of the same data type (e.g. nucleic acid sequences). A substring relation (*d*, *d'*) indicates that the DNA sequence *d *is a fragment of the longer DNA sequence *d'*. Of special interest are substring relations that place a fragment *d *near a coding region of *d'*. If *d *is no more than 50 base pairs away from overlapping a coding region of *d' *that encodes for protein *p*, then we say that *d *is linked to *p *(the strict threshold of 50 base pairs was chosen to ensure high quality, however, as Figure 3 shows, more permissive thresholds can be used to extend the set of links formed between DNA and protein sequences).

##### The 'similarity' relation

The similarity relation is one of the most fundamental relations in biology, frequently used for functional inference. Biozon computes and stores similarity relationships between proteins based on sequence, structure or expression profiles. The integration of similarity data enables the propagation of information from well-studied entries to uncharacterized ones.

Biozon contains pairwise similarities for about 2,000,000 sequences, which were computed using BLAST [29], resulting in a total of about 6.5 billion significant pairwise similarities (with *evalue *< 0.1). These similarity relations are used to extend the mappings from ESTs to proteins, thus increasing the set of functional descriptors that can be associated with an EST. The great advantage of the similarity relations of Biozon is the scalability and accessibility. Since EST analysis requires expensive database searches to search for possible protein products, it is difficult to scale existing methods for EST analysis to large libraries. By materializing similarity data, knowledge propagation in Biozon becomes immediate, thus facilitating the task of function assignment.

### Target proteins

A biologist might be interested only in ESTs that are linked to a specific biological system. To address this need, the EST2Prot system can be queried with respect to specific biological descriptors. The system collects a set of target proteins with relevant functions and reports the ESTs which map to at least one target protein. We define our target proteins by target descriptors, which are based on GO terms [30] and SwissProt keywords [22]. SwissProt keywords are descriptors that are associated with proteins based on manual curation. These keywords have been used in many studies to automatically annotate proteins or assess the biological function of protein clusters (e.g. [31,32]). The Gene Ontology (GO) functional descriptors are obtained from the GO database [30]. GO terms are organized in an acyclic tree-like graph where a node's parent represents a property that is more general than the node's property. However, unlike a tree form of a graph, in the GO graph it is possible to have more than one path leading from the root to a node. Also, a protein may be assigned more than one GO term, each one on a different branch of the graph (the different branches represent different groups of properties). GO terms in Biozon were collected from multiple sources, downloaded from the GO consortium website and extracted from databases such as UniProt. A total of 1,111,272 proteins in Biozon can be associated with GO terms in release 2.2. (Since protein databases contain many similar and almost identical proteins, the number of functionally *different *proteins with GO terms is obviously smaller).

### User interface

Given an EST (a GenBank or RefSeq accession number) EST2Prot explores all possible paths leading from that sequence to protein products in the Biozon data graph. The user is presented with multiple pages that summarize the information and rank the proteins based on our confidence in the association (depending on the type of the path). The first page provides the entry point to the Biozon data graph for the query EST and each page is linked to other pages with increasingly detailed information on the mapped proteins. For more information on the webserver see the Appendix (Additional File 1).

## Results

### Statistics

We analyzed in detail 185,543 UniGene clusters of Mouse and Human that were available as of April 2004 (105,680 human clusters and 79,863 mouse clusters). These clusters contain a total of 7,602,768 nucleic acid sequences, of which 125,235 are encoding sequences (i.e. sequences that contain a coding sequence that can be completely and directly mapped to an amino acid sequence). These encoding ESTs can be mapped directly to proteins using type I paths in Biozon. A total of 37,509 UniGene clusters contain at least one encoding DNA sequence.

Of the 185,543 UniGene clusters, 77,501 are associated with proteins by the UniGene team (type 3 paths). These clusters account for 7,196,998 of the 7,602,767 EST sequences (94.6%). By considering also direct relationships that are formed by coding sequences (type 4 paths), Biozon maps 79,760 clusters to proteins. Including also type 5 paths (using substring relations) results in 79,823 mapped clusters. It should be noted that the contributions of the Biozon-based paths of type 4 and 5 are substantial in and of themselves. For example, 37,658 clusters can be mapped based on class 4 paths alone and 13,370 clusters can be mapped based on type 5 paths alone. The latter is the result of 249,393 substring relations that we detected in the Mouse genome (involving 111,816 unique substring ESTs). These substring-superstring relations establish 169,480 relations between ESTs and proteins. [This multiplicity is characteristic of the data. If the EST is inside the coding region of a longer superstring, then the distance from the coding region is defined as zero. Sometimes, there might be multiple coding regions in the same DNA sequence containing that EST sequence. In these cases, the EST will be mapped to multiple proteins. Also, the EST will be mapped to multiple proteins if it is a substring of multiple DNA sequences, near coding regions.] The additional paths that Biozon explores naturally increase the number of ESTs that can be mapped to proteins. Moreover, these mappings are of high quality as they do not rely on cross-links by identifiers but rather on computationally validated transformations and relations. The most substantial difference between UniGene and the EST2Prot system lies in the number of paths formed and the number of proteins that can be mapped to EST clusters. When using only UniGene, there are 248,367 relations between UniGene clusters and protein sequences. With Biozon, the number of relations increases by 36% to 338,775 (even before considering similarity relationships) thus enriching the protein information significantly.

Interestingly, the 248,367 proteins that are mapped to 77,501 clusters by UniGene (type 3 paths) are reduced to only 49,412 *unique *protein sequences (less than 20% of the original set). Moreover, 38,534 proteins are mapped to at least two clusters. The most extreme case is of PIR protein S12207, with Biozon DocID 44431 [We refer to entities using their unique and stable Biozon 'DocID'. To view an entry with DocID *x*, follow the URL: http://biozon.org/Biozon/Profile/x]. that is mapped to no less than 1624 clusters. Similarly, Genpept protein GI:3355742 (Biozon DocID 69611) is mapped to 185 UniGene clusters. These numbers suggest very high redundancy and overlap between UniGene clusters, which is surprising given that clusters are expected to correlate with different genes.

Since Biozon adopts the UniGene clusters as is, the redundancy is also inherent to our mappings. However, the redundancy level is much lower and the 341,560 proteins that are mapped using paths of type 3, 4 and 5 are reduced to 136,635 unique proteins (more than 40%, compared to 20% with UniGene).

It should be noted that in some cases UniGene clusters "physically" overlap, as is the case for UniGene clusters Mm.334174 and Mm.247762. A total of 127 human clusters overlap with other human clusters, and 111 mouse clusters overlap with other mouse clusters. This can happen when multiple ESTs with different accession numbers are actually identical. Since Biozon employs a non-redundant object model, these identical sequences are mapped to the same nucleic acid sequence object in Biozon. It is unclear why these ESTs are clustered in UniGene to different clusters, whether the ESTs have different locations (i.e. the clusters correspond to paralogs), and if the UniGene clustering algorithm considers the location when grouping ESTs into clusters.

### Examples

As part of a study of pathways in the mouse olfactory system we were interested in identifying mouse ESTs whose protein products have brain-related functions from approximately 50,000 mouse ESTs provided by NIA and BMAP [33]. As target keywords and GO terms we chose those containing the text "brain," "nerv," or "neuro" anywhere in the keyword's or GO term's description. We also eliminate by hand keywords describing irrelevant diseases. Our target proteins are simply those described by the target keywords and GO terms (the complete list is available at [34]).

Of the 50,795 sequences in BMAP, 34,579 can be mapped to 30,463 proteins using UniGene alone. With the EST2Prot system, Biozon maps 35,185 ESTs to 56,848 proteins (87% increase). As many as 8,834 ESTs (17.39%) can be mapped to target proteins. When similarity data is considered the number of ESTs that can be mapped to target proteins more than doubles to 23,358 (45.98%).

To demonstrate the utility of the Biozon EST analysis tool, we used it to determine the potential function of ESTs identified in a microarray-based screen. This screen was designed to identify genes involved in axon pathfinding and target recognition in the mouse olfactory system. In our preliminary studies we identified thirteen genes that could potentially play key roles in this process. However, eight of these were ESTs with no known function or annotation. Biozon was able to find matches for four of the eight ESTs. Interestingly, one EST (AI843903) that is classified to a UniGene cluster of unknown function (as of October 2005) was predicted in Biozon to have similarity to protocadherins. Protocadherins are members of the cadherin superfamily, and are thought to play key roles in axon guidance, target recognition, and synaptogenesis [35]. Our preliminary results suggest that this EST is indeed a member of the cadherin superfamily, and may therefore be involved in mediating target recognition within the olfactory system. Without such annotation, this EST would likely have been ignored, and a valuable potential guidance cue may not have been recognized. Other ESTs (such as CX243176) that belong to the same UniGene cluster as AI843903 are also linked with protocadherins in Biozon, through similar paths, although neither UniGene nor Entrez suggest such links.

## Conclusion

To allow biologists to exploit EST libraries more efficiently and focus their search more effectively we developed an EST mapping system that identifies for each EST its most likely protein products. Our analysis relies heavily on NCBI's UniGene clustering, Biozon's infrastructure and the massive protein similarity data contained within. Our tool can help experimental biologists filter a large collection of nucleic acid sequences and predict which sequences are germane to a given biological system.

As an extensive and established EST analysis tool, UniGene is our main source of information. Biozon augments this data with other datasets that are extracted from multiple databases. For example, UniGene uses only 8 model organisms to map EST clusters to proteins, while Biozon uses protein sequences from thousands of fully or partly sequenced genomes, in addition to a myriad of other biological information on relationships between biological entities to establish multiple paths between EST sequences and protein sequences. Biozon also analyzes sequences in the NCBI EST database (dbEST) that are excluded from the gene-oriented UniGene, such as rRNA and mitochondiral sequences.

The retrieval of this information is done in real-time by traversing paths in the data graph. This is made possible because of the graph-schema of Biozon that was designed to handle large-scale integration of dynamically changing biological data, where all datasets are compiled into a single tightly-connected graph. Since the data in Biozon relies on physical properties (e.g. the actual sequences or sets thereof) rather than just database cross links, the graph-link structure is of high quality. Moreover, the Biozon database was designed to sustain frequent updates of its sources, and as such it ensures that the mapping utilizes newly sequenced DNA and protein sequences.

Biozon also strives for completeness, and the similarity data makes up for missing and inconsistent data. This has a major impact for example, when compiling the functional descriptors from the GO database. Since GO data is partial, and since it is derived from multiple sources, it is not necessarily coherent. Consequently, in many cases proteins that are very similar based on sequence are not necessarily associated with the same set of GO terms, and this can greatly reduce the effectiveness of the mapping. By incorporating similarity relations Biozon extends and enrich the set of functional descriptors that can be associated with a given EST.

The EST2Prot system can be accessed from the Biozon webserver. Information on the web tools is available in the Appendix (see Additional File 1).

## Availability and requirements

EST2Prot is available online at . Any user can upload and analyze her or his EST data through any javascript-enabled web browser such as Netscape, Mozzila or Explorer, and obtain the mapping in real-time.

## Authors' contributions

PS implemented the EST2Prot system and ran experiments over the mouse and human genomes. GY and DL conceived of the study. GY designed the system. DL participated in the analysis of the results. All authors contributed, read and approved the final manuscript.

## Supplementary Material

Additional file 1 Appendix The EST2Prot webserver. The appendix takes the reader through the main web pages that make-up the EST2Prot webserver, with explanations of the output format of each page and snapshots that exemplify the type of information provided. Click here for file
