# Supplementary material for: EST2Prot: Mapping EST sequences to proteins
Source: BMC Genomics. 2006 Mar 4;7:41. doi: 10.1186/1471-2164-7-41 (PMC1456965; doi:10.1186/1471-2164-7-41)
Supplement: Additional file 1 — Appendix The EST2Prot webserver. The appendix takes the reader through the main web pages that make-up the EST2Prot webserver, with explanations of the output format of each page and snapshots that exemplify the type of information provided. [file 1471-2164-7-41-S1.pdf]

Supplementary material for *EST2Prot: Mapping EST sequences to proteins*

Paul Shafer[1], David M. Lin[2] and Golan Yona[1,*]
[1] Department of Computer Science, Cornell University, Ithaca, NY
[2] Department of Biomedical Sciences, Cornell University, Ithaca, NY
*Corresponding author. Email: golan@cs.cornell.edu

1 Appendix - The EST2Prot webserver

The EST mapping system consists of 5 pages: the upload page, the summary page, the EST map page, the descriptor page, and the path page. The user starts with the upload page, which allows the user to submit ESTs for analysis. The user is then taken to the summary page, which summarizes each uploaded EST by displaying descriptors of proteins associated with it, and other pages with detailed information about the mapping. An overview of the webserver is given in Figure 1.

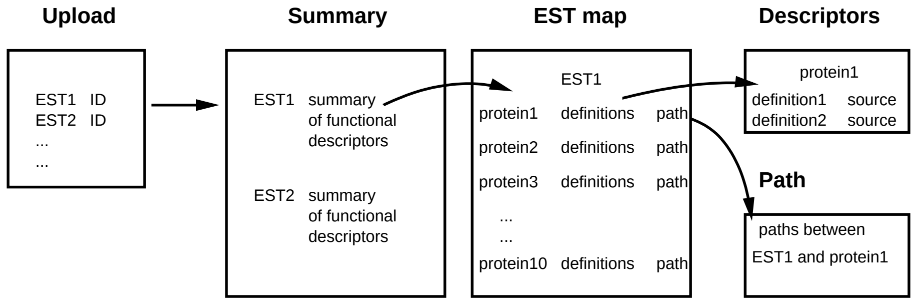

Figure 1: **The Biozon EST2Prot webserver**.

1.1 The Upload Page

The upload page (Figure 2) allows the user to upload a list of ESTs for analysis. This is done by specifying either the Genbank accession number or Genbank GI number of each EST. The user may upload a file of identifiers or type their identifiers into a text box.

1.2 The Summary Page

The *summary page* summarizes the possible functions of each uploaded EST (Figure 3). This page has four columns. The first column displays the Genbank identifier of the uploaded EST. If the identifier was found in Biozon's local copy of Genbank, the user may click on the identifier to view Biozon's record of the corresponding nucleic acid sequence.

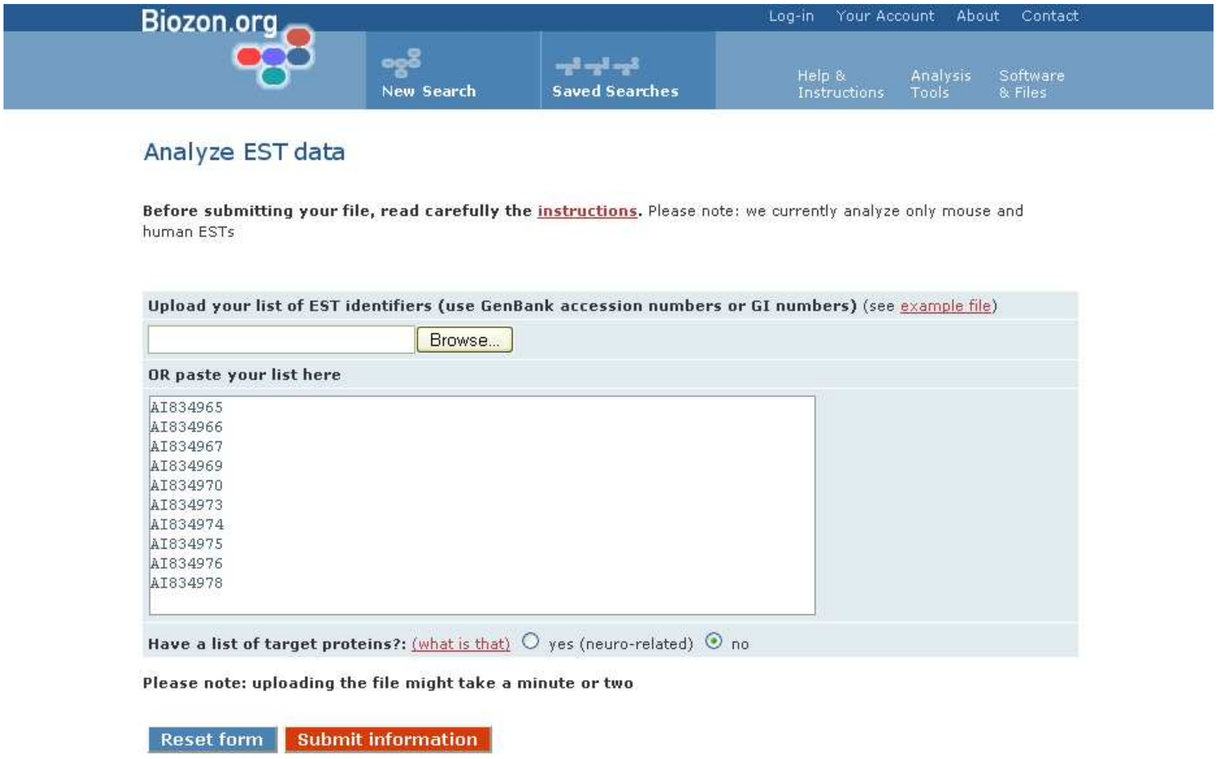

Figure 2: **The Upload Page**.

The second column displays definitions of proteins which are mapped to each EST. At most ten non-redundant definitions appear, and the number in parenthesis following each definition is the number of times that definition was observed. To facilitate the presentation of this information, we align the descriptions using a variation on a dynamic programming algorithm that considers the sentence structure as well as the actual descriptions when aligning descriptions (Yona & Leung, unpublished). Descriptions are then grouped based on their similarity scores. If "*(sim)*" follows a definition, then similarity data was used in the corresponding map.

The third column displays GO terms and Swiss-Prot keywords associated with the proteins mapped to each EST. Again, if "*(sim)*" follows a descriptor, then similarity data was used in the corresponding map.

In both the second and third columns, descriptors are displayed in order of map type. That is, descriptors of proteins mapped by type 1 paths appear first, type 2 paths appear second, and so on. Descriptors corresponding to similarity maps appear after direct maps and are also ordered by type.

The fourth column displays "yes" if the corresponding EST maps to a protein which is involved in an interaction and displays "no" otherwise. Similarly, if the proteins are on the list of target proteins then the corresponding column is marked.

1.3 The EST Map page

The *EST map page* displays more detailed information on each of the proteins mapped to a particular EST (Figure 4). This page has six columns. The first column displays the mapped protein's NR identifier. The

user may click on the identifier to view Biozon's record of that protein, containing information on the broader biological context of the protein (such as the DNA sequences that encode the protein, the interactions it is involved with, the structures it is linked to and the other entities it is similar to).

The second column displays the protein's primary definition and the third column displays the protein's descriptors. Clicking the "see more" link in either of these columns takes the user to the *descriptor page* where the user finds a comprehensive list of the protein's definitions, GO terms, and Swiss-Prot keywords.

The fourth column indicates whether or not the protein is involved in an interaction. The fifth column displays the type of the corresponding map, and the sixth column contains a link to the *path page* where the user finds the details of the corresponding map between the EST and the protein.

1.4 The Descriptor Page

The *descriptor page* displays all the definitions, GO terms, and Swiss-Prot keywords associated with a particular proteins (Figure 5). For each definition, the descriptor page displays the source database of that definition. The user may click on any of the displayed GO terms to view Biozon's record of the term and the corresponding graph. The descriptor page only displays GO terms actually assigned to the protein (not all ancestors of these GO terms). However, the parent GO terms can be viewed through the Biozon profile page of each GO term.

1.5 The Map Page

The *map page* displays the details of every map from the chosen EST to the chosen protein (Figure 6). The maps are displayed in order of their type, with type 1 maps appearing first, type 2 maps appearing second, and so on. Maps which use similarity data appear after direct maps and are also ordered by type.

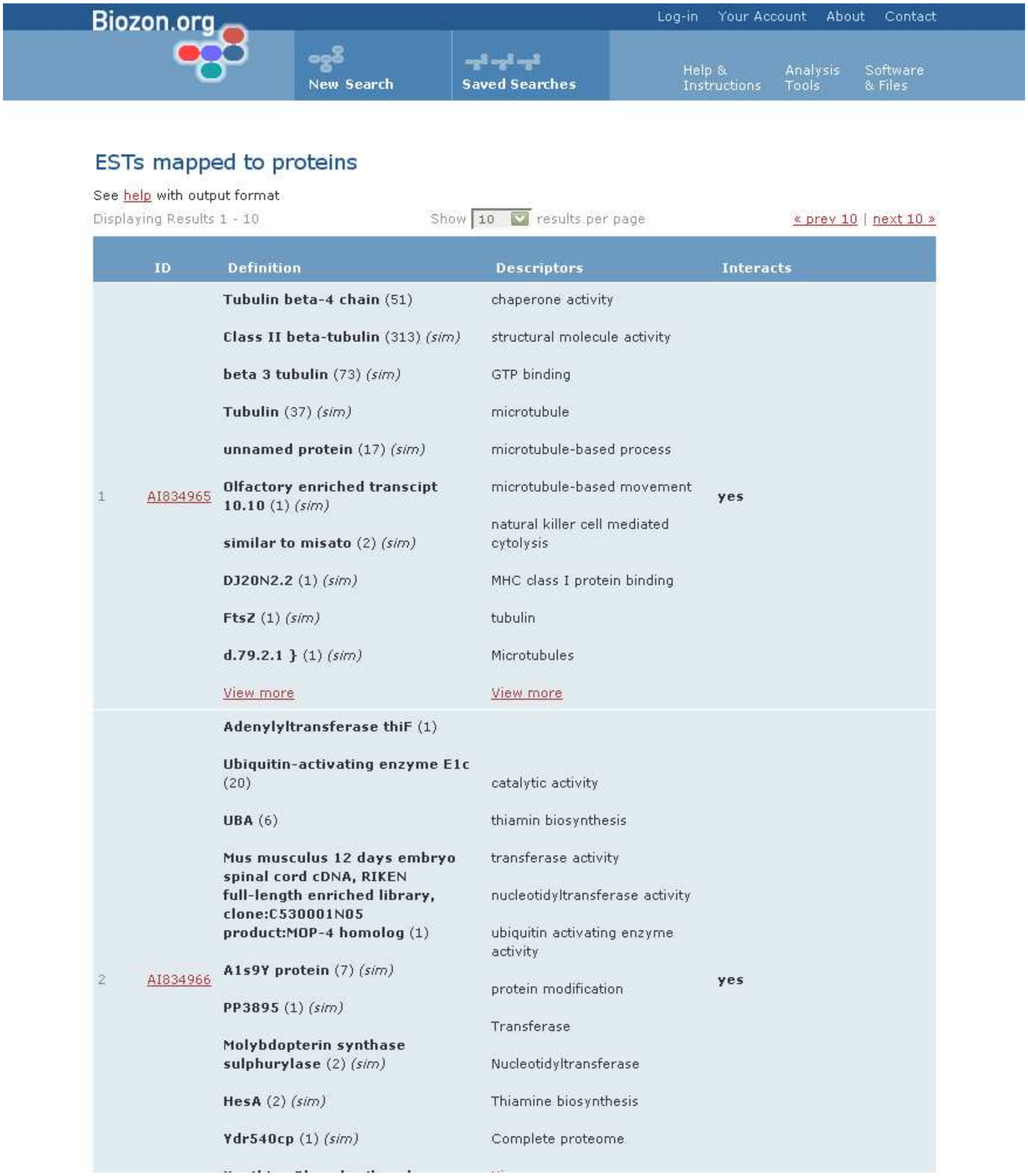

Figure 3: **The Summary Page**.

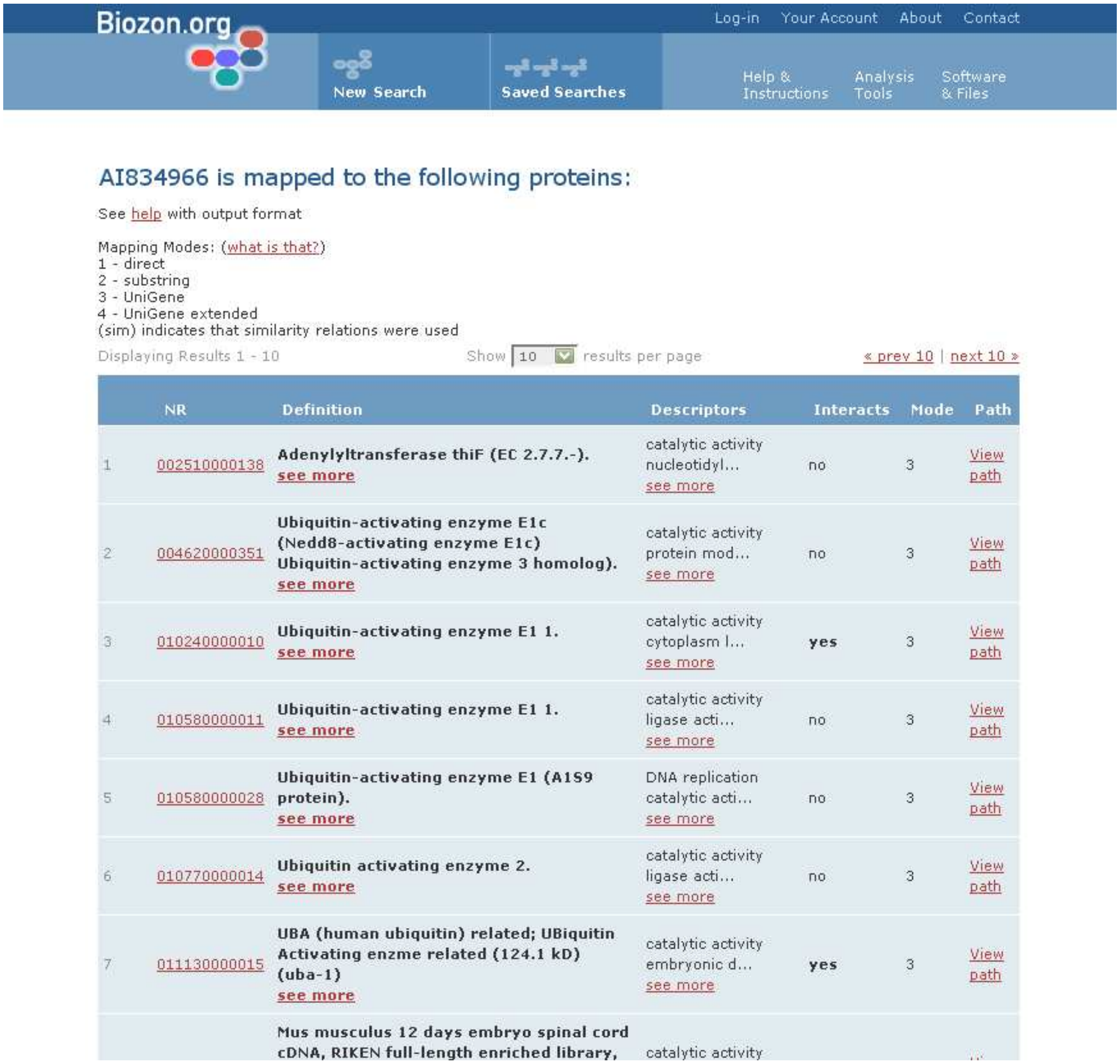

Figure 4: **The Map Page**.

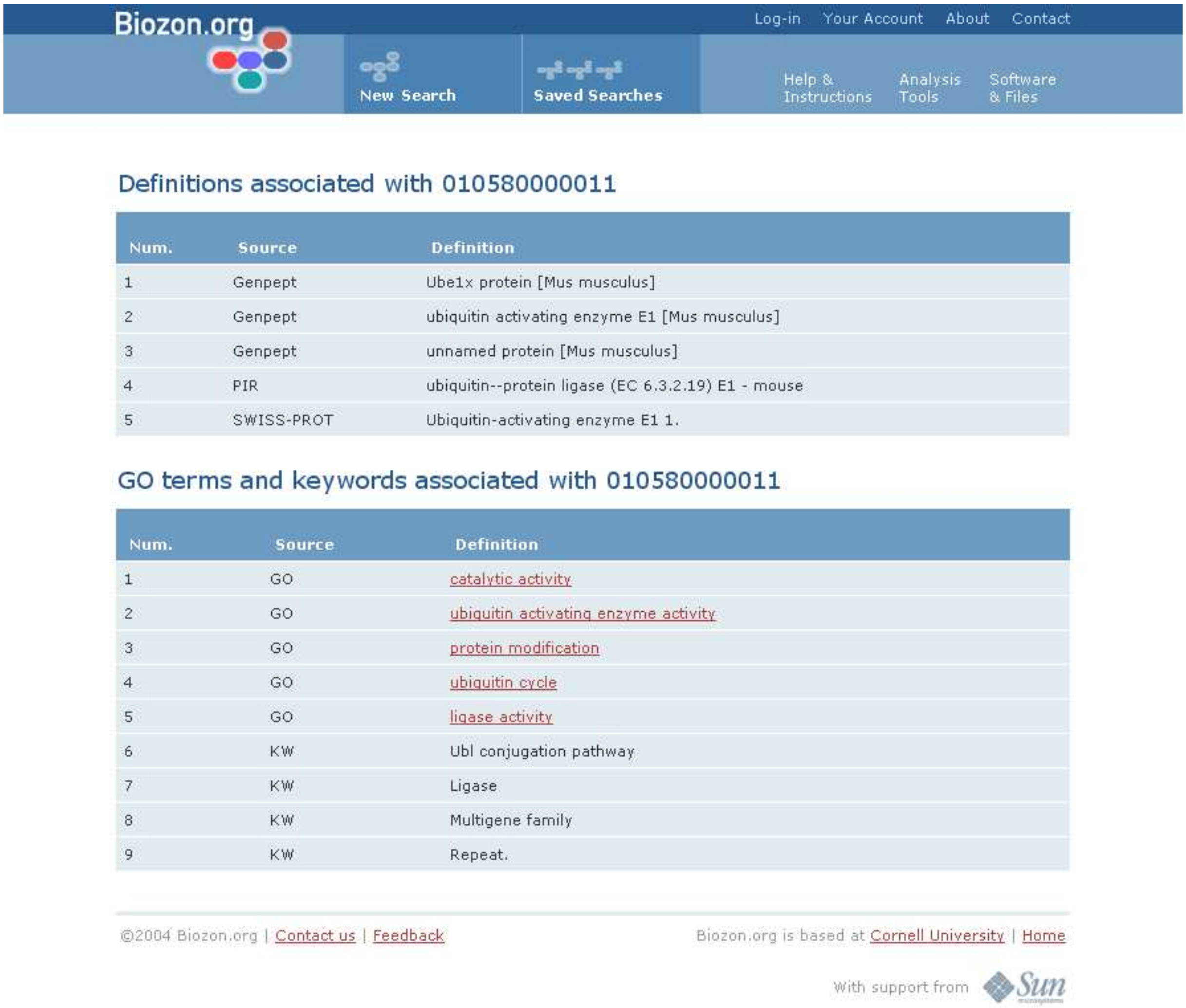

Definitions associated with 010580000011

Num.	Source	Definition
1	Genpept	Ube1x protein [Mus musculus]
2	Genpept	ubiquitin activating enzyme E1 [Mus musculus]
3	Genpept	unnamed protein [Mus musculus]
4	PIR	ubiquitin--protein ligase (EC 6.3.2.19) E1 - mouse
5	SWISS-PROT	Ubiquitin-activating enzyme E1 1.

GO terms and keywords associated with 010580000011

Num.	Source	Definition
1	GO	catalytic activity
2	GO	ubiquitin activating enzyme activity
3	GO	protein modification
4	GO	ubiquitin cycle
5	GO	ligase activity
6	KW	Ubl conjugation pathway
7	KW	Ligase
8	KW	Multigene family
9	KW	Repeat.

Figure 5: **The Descriptor Page**.

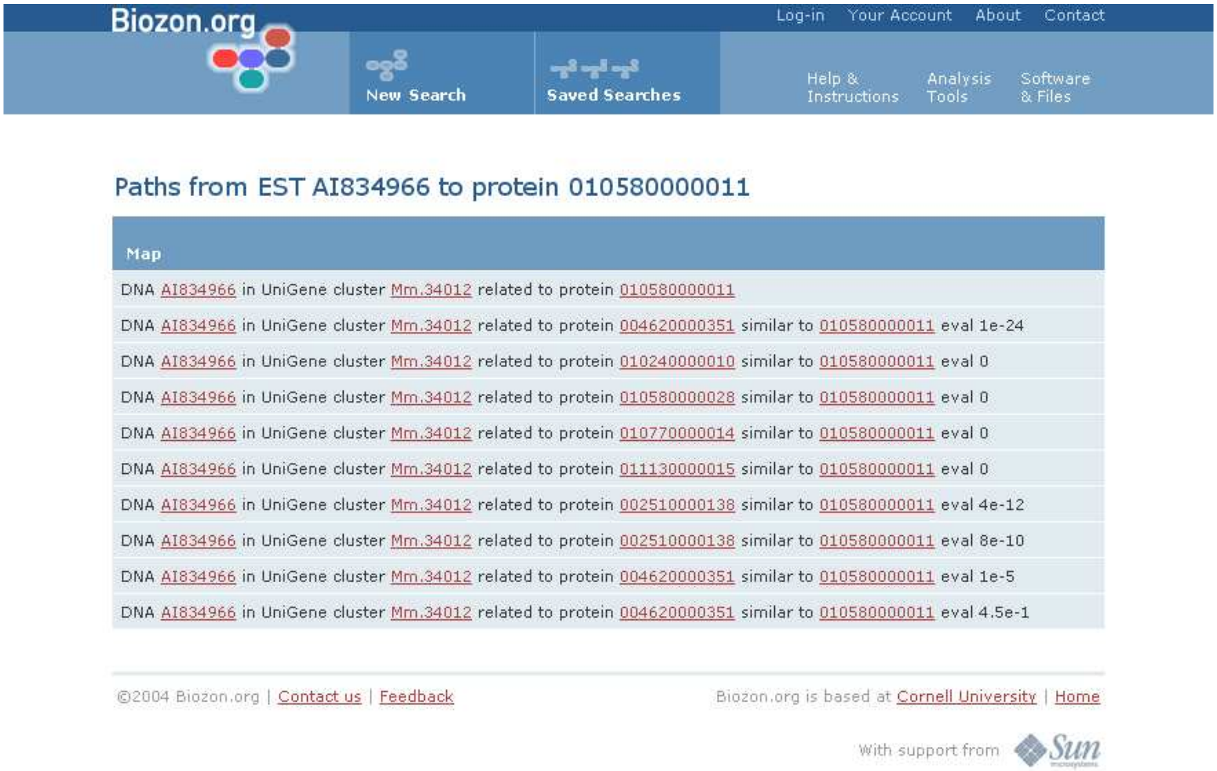

Figure 6: **The Path Page**.
